# The Detection of COVID-19-Related Multivariate Biomarker Immune Response in Pediatric Patients: Statistical Aspects

**DOI:** 10.3390/v17030297

**Published:** 2025-02-21

**Authors:** Michael Brimacombe, Aishwarya Jadhav, David A. Lawrence, Kyle Carson, William T. Lee, Alexander H. Hogan, Katherine W. Herbst, Michael A. Lynes, Juan C. Salazar

**Affiliations:** 1 Connecticut Children’s Medical Center, Hartford, CT 06106, USA; 2 Department of Pediatrics, UConn Health, Farmington, CT 06030, USA; 3 Wadsworth Center, New York State Department of Health, Albany, NY 12208, USA; 4 Department of Biomedical Sciences, College of Integrated Health Sciences, University at Albany, Rensselaer, NY 12144, USA; 5 Department of Molecular and Cell Biology, University of Connecticut, Storrs, CT 06269, USA

**Keywords:** artificial neural network, biomarkers, biplot, correlation, diagnostic testing, dimension reduction, immune system, logistic regression, MIS-C, principal components, SARS-CoV-2 infection

## Abstract

The development of new point-of-care diagnostic testing tools for the detection of infectious diseases such as COVID-19 are a key aspect of clinical care and research. Accurate predictive classification methods are required to correctly identify and treat patients. Here, the onset of multisystem inflammatory syndrome in children (MIS-C), a more serious form of COVID-19, was predicted in a pediatric population using a set of multivariate immunological biomarker expression values. A first-stage bivariate detection of statistically significant biomarkers was obtained from a chosen set of standard cytokines and chemokine biomarkers considered relevant to COVID-19-related infection and disease. To incorporate the observed correlation structure among the resulting set of significant biomarkers, dimension reduction was then applied in the form of principal components. A second-stage logistic regression model using a small number of the principal component variables provided a highly predictive classification model for MIS-C. The resulting model was shown to compare favorably with an artificial neural network-based predictive model.

## 1. Introduction

COVID-19 is an infectious respiratory illness that results in symptoms such as cough, fever, fatigue, chest pain, loss of taste or smell, etc., in children and adults [1]. The COVID-19 symptoms observed in most children tend to be milder compared to those in adults, but in rare cases, some children develop the more serious multisystem inflammatory syndrome in children (MIS-C). This condition involves the severe inflammation of multiple organs approximately four-to-six weeks after being infected with the SARS-CoV-2 virus [2,3]. A child’s immune profile in response to SARS-CoV-2 infection may enable accurate prediction of a MIS-C occurrence and help guide treatment strategies [4,5]. Although MIS-C is rare, it is a serious medical condition that resembles Kawasaki disease (KD) and other multisystem inflammatory disorders [6]. The physiological processes leading to MIS-C remain an area of investigation [7].

The development of new point-of-care assessment diagnostic tools for the detection of infectious diseases such as COVID-19 are key aspects of clinical care and research [8]. The development of a new diagnostic testing chip-based approach to incorporate a large number of biomarker expressions and use these to identify the onset of related infection or condition, irrespective of patient clinical profile, was the context of our study. A Luminex xMAP^®^ multiplex assay was applied and used to determine a set of statistically significant biomarkers to include on the chip in relation to detecting the onset of MIS-C.

As MIS-C is a novel, COVID-19-related condition, immunologically based analysis will initially require the assessment of a wide-ranging set of potential biomarkers—using a Luminex xMAP^®^ multiplex assay—that may be expressed in relation to the condition. In terms of related statistical analysis, this can lead to some irrelevant biomarkers being included in the initial testing stage, as well as a large number of statistically significant biomarkers. In this context, while immunological studies typically focus on single comparisons of individual biomarkers between cases and controls, there is also a need to recognize that many of the significant immunological variables observed will be correlated, reflecting the systemic nature of the immune response.

In such settings, when modeling, it will often be the case that many biomarkers combinations will achieve very similar levels of model fit to the data, or the statistical models may not converge [9]. This is most easily detected using stepwise techniques to fit the model of interest, as the backward and forward selection procedures will not give the same results and will show various signs of instability (for example, selecting variables with high *p*-values due to the correlated nature of the data) [9]. Challenges arising when fitting linear models with correlated explanatory variables are well known [9], and, in cases where the response of interest is continuous, these have led to the development of methods such as partial least squares, factor analysis, ridge regression, and Stein estimation, each with many modifications and complexities [10]. Dimension reduction in the form of singular value-based decomposition (SVD) or related principal components analysis (PCA) is often a key step in these analyses [11].

Machine learning models, notably artificial neural networks (ANNs), offer a competing paradigm for the fitting (training) of predictive classification models [12]. These models are essentially black boxes, providing predictive accuracy but limited understanding of how individual variables—for example, immunological biomarkers for MIS-C—contribute to overall predictive accuracy. However, it is often useful to compare the predictive accuracy of such black box models with more standard approaches to obtain a measure of the possible levels of predictive accuracy. In settings where there are specific data structures present, such as clusters of correlated variables, interpretable statistical models may provide similar levels of accuracy and a greater understanding.

In this paper, the focus was on developing a predictive classification model for MIS-C patients versus controls in a small-to-moderate sample size using the systemic, correlated multivariate nature of the immune system response. A first-stage initial selection of significant individual biomarkers, corrected for multiple comparisons, was used to identify a subset of statistically significant but correlated variables. The PCA method was then used to incorporate the observed correlation structure to both reduce the dimension of the problem and yield interpretable summary PCA variables. A second-stage logistic regression based on the PCA variables was then applied to obtain a predictive classification model for MIS-C versus controls. This was also compared to a supervised ANN-based classification model based on the set of statistically significant variables without principal component adjustment.

## 2. Methods and Materials

The results presented here used clinical and laboratory data collected under a study funded by the National Institutes of Health’s Rapid Acceleration of Diagnostics [6]; Predicting Viral-Associated Inflammatory Disease Severity in Children with Laboratory Diagnostics and Artificial Intelligence (PreVAIL kIds). Data were collected as part of an NIH funded RADx/Prevail Kids [13] project that seeks to develop a chip-based technology based entirely on a large number of chosen immunological biomarkers [14] for the on-site detection of COVID-19, MIS-C, and KD cases. Comprehensive biomarker discovery was used to characterize the clinical and laboratory spectrum of children and adolescents with mild, moderate, and severe SARS-CoV-2 infection, as well as MIS-C.

### 2.1. Patient Groups

Subjects aged from birth to ≤21 years old who had serum samples were included. Participants were classified by a panel of three pediatric physicians (one pediatric hospital medicine and two pediatric infectious diseases physicians) into one of five diagnostic cohorts as per the study protocol: (i) positive for SARS-CoV-2 infection per antigen or PCR testing and hospitalized for COVID-19 symptoms (COVID-19+); (ii) hospitalized and meeting the Center for Disease Control’s criteria for MIS-C; (iii) hospitalized and meeting the Center for Disease Control’s criteria for KD; (iv) hospitalized for respiratory viral illness (Viral); and (v) controls undergoing routine ambulatory surgery (non-emergency surgery that does not require an overnight stay in the hospital) for conditions unrelated to COVID-19 or an inflammatory condition. Blood was collected at enrollment, and the relevant clinical information and serum samples were processed. Serum was isolated by resting the blood for approximately 30 min after collection to allow for coagulation. The non-coagulated part of the blood was transferred into a 15 mL tube and centrifuged at 1000× *g* for 15 min. Supernatant was then transferred into a new tube, and centrifuged at 1000× *g* for 5 min. Supernatant was aliquoted into 200µL cryo vials, and stored at −80^o^. Blood was collected in 21% (14/67) of patients (MIS-C and KD) prior to their receiving IVIG. The onset of MIS-C or KD symptoms ranged from three to twenty days (median = 6) prior to blood collection. The focus of the analysis here was on differentiating the MIS-C group from controls. All required ethical and IRB (#21-004) approvals were obtained.

### 2.2. Biomarker Variables

All serum samples were analyzed for levels of a battery of 26 standard immunological cytokines/chemokines, chosen by participating microbiologists, pediatric clinicians, and infectious disease researchers in the research group. A standard Luminex xMAP^®^ multiplex assay was used. Cytokines/chemokine levels in the serum samples were measured in duplicates using the Luminex^®^ 200^™^ (Luminex Corporation, Austin, TX, USA) instrument and Milliplex^®^MAP (Merck KGaA, Darmstadt, Germany) kits from EMD Millipore (Cat #HSTCMAG-28SK, HCYTA-60K, HCYP2MAG-62K, and HCYP4MAG-64K) according to manufacturer’s protocol.

Cytokine/chemokine levels in the serum samples obtained during the initial admission were measured in duplicate using the Luminex^®^ 200^™^ instrument and Milliplex^®^MAP kits from EMD Millipore (Cat #HSTCMAG-28SK, HCYTA-60K, HCYP2MAG-62K, and HCYP4MAG-64K) according to the manufacturer’s protocol. A 96-well plate provided with the kit was first washed with 200 µL of wash buffer. The wash buffer was then discarded, and 25 µL of serum sample was added to the plate in duplicate along with 50 µL of standards and controls, which were provided with the kits. Assay buffer (25 µL) was then added to the sample wells, followed by 25 µL of premixed magnetic beads (provided with the kit) to each well. The plate was covered with a dark lid and placed on a plate shaker (200 rpm on a Barnstead 4625 Titer plate shaker) overnight at 4 °C in a dark room. The following day, the plate was washed three times with 200 µL of wash buffer using BioTek ELx405^™^ (Agilent Technologies, Santa Clara, CA, USA) microplate washer with magnetic capture. Following washing, detection antibodies (50 µL) were added to each well, and the plate was incubated for 1 h on a plate shaker covered with a foil. Streptavidin–phycoerythrin (50 µL) was added to each well, and the plate was incubated for 30 min on a plate shaker covered with foil. Lastly, the plate was washed three times with 200 µL of wash buffer using BioTek ELx405^™^ microplate washer and analyzed using the Luminex^®^ 200^™^ instrument (calibrated each week with Luminex 200 Calibration and Performance Verification kits: Cat # LX2R-CAL-K25, LX2R-PVER-K25) with 150 µL of sheath fluid present in each well. Standard curves were generated using the Luminex xPONENT^®^ software (version 4.3), and the concentrations of cytokines/chemokines in the serum samples were calculated using the standard curves in pg/mL [15].

The variables chosen and measured were chemokine (C-C motif) ligand 20 (CCL20), CCL3, CCL4, CX3CL1, CXCL11, granulocyte–macrophage colony-stimulating factor (GMCSF), interferon IFN-α2, IFN-β, and IFN-γ, and interleukin IL-1β IL-2, IL-4, IL-5, IL-6, IL-7, IL-8, IL-10, IL-12(p70), IL-13, IL-15, IL-17A, IL-18, IL-21, IL-23, IL-33, and TNF-α.

### 2.3. Multivariate Data Analytic Methods

While measured in the laboratory one variable at a time, many immune variables are often found to be highly correlated. To incorporate this information and accommodate these data patterns, a multi-stage approach was employed to detect statistically significant variables and use them to develop a predictive classification model for differentiating MIS-C subjects from controls. One-way analysis of variance (ANOVA) with Tukey adjusted pairwise differences [9] was utilized to identify a set of statistically significant biomarkers differentiating MIS-C from controls.

Once the set of statistically significant biomarkers was determined, the correlation structure was examined, and principal component analysis (PCA) was used to further reduce the dimension of the data. PCA variables are uncorrelated by construction and provide distinct dimensions related to the eigenvector-based decomposition of the sample correlation matrix of the set of statistically significant variables. Often, only a few PCA variables are required to capture a large proportion of the variability in the relevant data, and these are interpretable in relation to the original set of biomarkers.

Here, given a set of eighteen significant variables, many of which are highly correlated, principal components analysis used the eigenvectors and eigenvalues of the observed sample correlation matrix, subject to order restrictions, to provide a new set of eighteen uncorrelated principal component variables. Each variable was a weighted sum of the original variables, with the eigenvalue related to each principal component giving the contribution of each variable to total variation.

Graphical biplot and cluster analysis [16] were also used to examine the correlation structure of the set of statistically significant immunological biomarkers and the degree to which the first two PCA variables provide useful classification. A second-stage predictive classification logistic regression model, using the PCA variables, then provided a fitted model. This gives diagnostic measures of model sensitivity, specificity, positive predictive value (PPV), negative predictive value (NPV), overall classification error, receiver operating characteristic curve (ROC), and area under the ROC curve (AUC).

For comparative purposes, a supervised ANN predictive model used the set of statistically significant variables. The model was convergent for several levels of complexity; the number of hidden layers set equal to moderate levels of 18 and 36. Predictive classification diagnostic results and ROC curves were obtained and compared with the principal components-based approach. All calculations were carried out using the R (version 4.2.1) and STATA (version 16) statistical packages. *p*-values < 0.05 were considered significant, unless subject to multiple comparison correction.

## 3. Results

Included in the study were 246 subjects. Demographics are found in Table 1.

Twenty-six standard immunologic biomarkers (cytokines and chemokines) were initially assessed for differences between the five patient groups. One-way ANOVA using Tukey pairwise comparisons (correcting for multiple comparisons across the five patient groups) was also used as an element of the initial analysis, allowing for detection of biomarkers having statistically significant differences between the MIS-C (n = 48) and control (n = 69) groups, subject to adjustment for multiple comparisons. Eighteen of the twenty-six biomarkers showed significant adjusted *p*-values when comparing the two groups. These were as follows: CCL4, CXCL11, GMCSF, IL-1β, IL-2, IL-4, IL-5, IL-6, IL-7, IL-8, IL-10, IL-13, IL-15, IL-18, IL-21, IL-23, IL-33, TNF-α (see Table 2).

**Figure 1:**
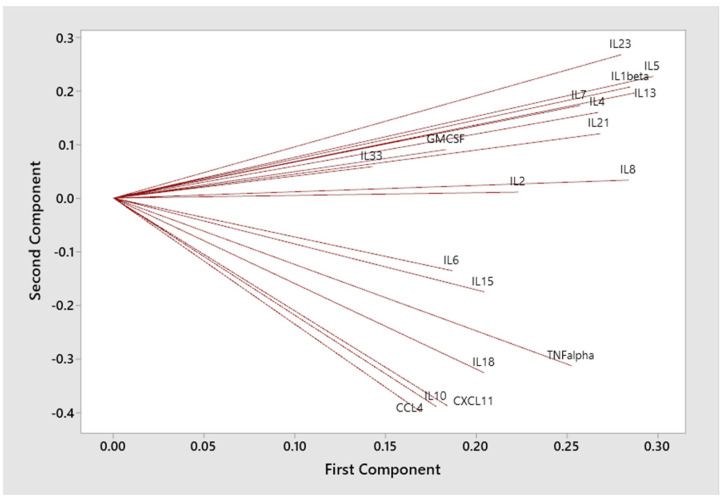
Biplot showing the loadings and related correlation structure of significant biomarkers.

**Figure 2:**
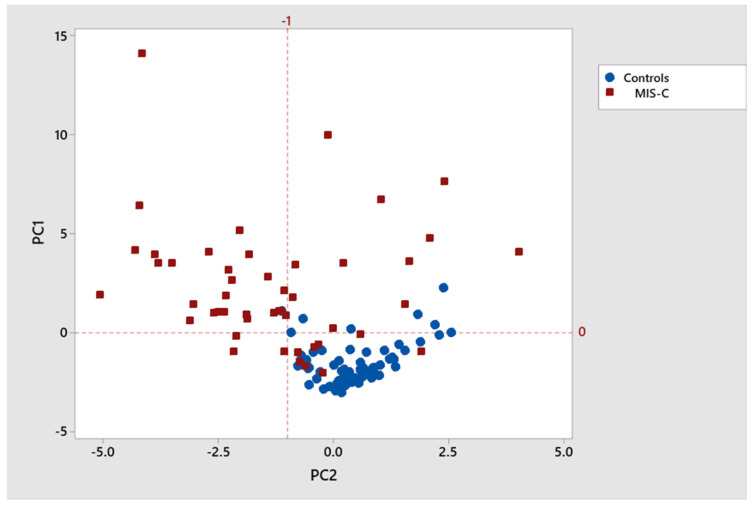
MIS-C versus controls principal components-based cluster plot.

Of the original 18 significant biomarkers, Table 3 shows a subset of 11 biomarkers having pairwise Pearson correlations > 0.5 (bolded). This clustering is also visually depicted in a biplot showing the correlation structure and related clustering (see Figure 1 and Figure 2). Standard logistic regression for MIS-C versus control using the original 18 statistically significant analyte variables did not converge.

The PCA approach and related dimension reduction was then applied to the set of 18 statistically significant variables, using their correlation matrix. This produced a new set of 18 uncorrelated principal components variables, each of which are weighted averages of the original 18 significant biomarkers. These weights are referred to as loadings. Typically, only the first few PCA variables are used as they often account for a significant proportion of the total variation in the data. The first two PCA variables here accounted for 54% of total variation and showed a useful level of discrimination between those with and without MIS-C (see Figure 2). In terms of their loadings, key biomarkers included IL-4, IL-6, IL-8, IL-10, IL-13, CCL4, CXCL11, and TNF-α (see Table 2).

**Figure 3:**
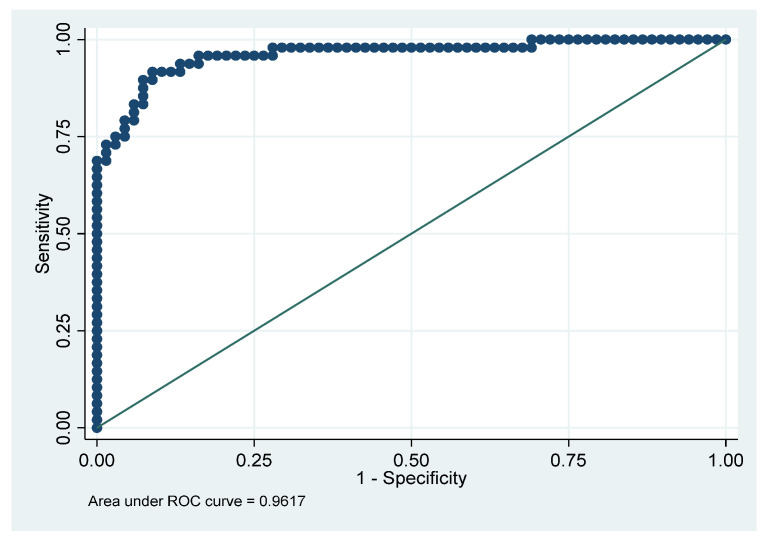
Logistic regression ROC curve.

A logistic regression model was developed using only the first two PCA variables (Table 4) to predict a binary variable (MIS-C = 1; controls = 0). The respective ROC curve is given in Figure 3. The fit of the model was very good, with the following characteristics: sensitivity = 83%; specificity = 94%; false+ = 9%; false− = 11%; correctly classified = 90%; and AUC = 96%.

**Figure 4:**
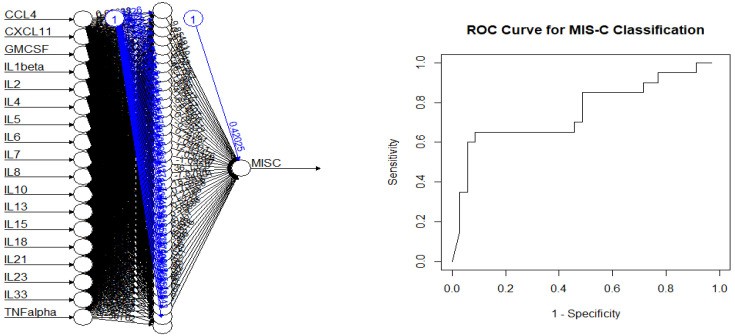
Artificial neural network and ROC curve (H = 36).

For comparison, an ANN model, using the R neuralnet function was trained to the data using a 50/50 random split for generating the training and testing components of the dataset. All eighteen statistically significant variables were used, without principal component adjustment, with the number of hidden layers set at H = 18 and 36. The results are shown in Table 5 with the respective ROC curve for the H = 36 case given in Figure 4.

The classification diagnostics here show greater predictive ability occurring in the overall fit of the model, with the H = 36 model having the following diagnostic characteristics: sensitivity = 65; specificity = 89%; false+ = 11%; false− = 35%; correctly classified = 80%; and AUC = 73%. For this dataset, logistic regression using principal components dimension reduction reflecting correlation structures compared well with the supervised ANN-based model.

## 4. Discussion

There is often a need to understand biomarker activation as a component of the immune response to an infection or related condition. When this is a new infectious agent or condition, this often leads to a wide range of possible biomarkers being examined in a small to moderate sample size and results in a large number of correlated, significant variables. This is usually presented in terms of bivariate comparisons of each individual biomarker between cases and controls, with pairwise correlations ignored.

Standard multivariate analyses using unmodified sets of correlated variables can be unstable, and many methods are available to incorporate this structure to varying degrees, especially if the response is continuous. Factor analysis, partial least squares, Stein estimation, and ridge regression [10] are all means of dealing with correlated sets of predictive variables in developing multivariate predictive models. Here, the principal components method was employed to derive a second-stage set of uncorrelated variables, each a weighted sum of the original set of significant variables. This provided an interpretable set of variables with which to accurately classify MIS-C versus control patients.

Machine learning models, notably ANNs, offer a competing paradigm for the fitting (training) of predictive classification models. However, they are known to be of limited predictive value in smaller samples [17]. Here, the application of an ANN model for the set of significant explanatory variables, using moderate levels of complexity, did not yield an improvement to the two-stage logistic regression model using PCA-based variables.

The PCA-based approach, along with the underlying scientific and medical insight, allowed for the interpretation of the final set of variables to be included. The initial use of one-way ANOVA to screen for individually significant variables allows for the incorporation of effect sizes and initial multiple comparison issues. The resulting sample correlation matrix provided an additional source of information, and the observed high levels of correlation for the significant variables were decomposed using principal components, with the first two PCA variables giving a very useful second-stage predictive classification logistic regression model. Note that the use of continuous explanatory variables in logistic regression—a generalized linear model—is acceptable, especially if the focus is an overall predictive model. Moreover, in the context of the logistic regression predictive model, normality of the principal component variables is not required [9].

The set of initially significant biomarkers, considered as a correlated set of immunological variables, supported the development of a highly predictive classification model. Key biomarkers identified here include type 2 cytokines IL-4 and IL-6, affecting B cell stimulation, growth, and differentiation factors, and reflecting their role in humoral responses [18,19]. IL-10 is often considered an anti-inflammatory cytokine [20], and expression increases to regulate inflammation [21]. Chemokines CCL4 and CXCL11 affect innate cell activation and recruitment [22]. Higher levels of cytokines (IL-8, IL-13) are known to influence eosinophil activity [23,24]. IL-13 functions similarly to IL-4 as both cytokines have a common receptor (IL-4Rα) for signaling cells [25].

Recent work applying logistic regression models to various viral infections, including COVID-19-related infection and related conditions, can be found in [26,27] and the references therein. Discussion of the effects of correlated explanatory variables on logistic regression can be found in [28].

Practical issues often affect the interpretation of immunological biomarker results. This study primarily included children hospitalized for MIS-C and KD, for which intravenous immunoglobulin (IVIG) is standard treatment. However, it is not standard for any of the other study groups. The effects of IVIG on the levels of biomarkers in the blood are not clear. Blood was collected in 21% (14/67) of patients (MIS-C and KD) prior to their receiving IVIG. The onset of MIS-C or KD symptoms ranged from three to twenty days (median = 6) prior to blood collection. Recent work has linked IL-6 expression to IVIG non-response in KD patients, a condition closely related in symptomology to MIS-C patients [29]. Given these findings, the effect of the IVIG treatment on biomarkers measured in the sera may be limited but also may have had some diminishing effect on biomarker expression.

As the study design reflected the goal of developing a new chip-based technology based entirely on biomarker-based information to inform clinical diagnoses, the choice of information and data for analysis was limited. Aspects of biomarker immune response related to individual differences, medical and family history, associated or unassociated comorbidities, differential diagnosis, the stage of the clinical condition, medications, and forms of interventions/treatments may also be relevant to the presence of MIS-C and were not included here due to our focus on the development of a serum-based diagnostic testing technology.

Note that other approaches to the definition of controls and related data are possible. In the context of a broader study design, the reported analysis focused on the comparison of pediatric MIS-C patients versus controls recruited primarily in a pediatric hospital. Moreover, not all data will reflect the presence of correlated structures among biomarkers, allowing for the application of principal components-based dimension reduction. In many settings, a trained, supervised ANN model may provide highly accurate predictive classification (see, for example [30]).

Immunological signatures are important to understanding the ontogeny of innate and adaptive immune responses following exposure to SARS-CoV-2 and other infections. These immune profiles generate very extensive phenotypic and functional profiles, which can be analyzed using the method outlined here to determine a set of biomarkers that can predict antibody or T cell responses related to MIS-C or other types of infections and related medical conditions. Future work will include the development of biomarker-based diagnostic testing and related predictive statistical and machine learning/A.I. models for differentiating MIS-C from KD. Note that the example dataset examined here can be found as part of the RadX Data Hub [31].

## 5. Conclusions

An accurate, interpretable, predictive classification model for MIS-C patients versus controls in a small-to-moderate sample size, using the systemic, correlated multivariate nature of the immune system response, was obtained without the inclusion of clinical variables. A first-stage initial selection of significant individual biomarkers, corrected for multiple comparisons, was used to identify a subset of statistically significant but correlated variables. The PCA method incorporated the observed correlation structure to both reduce the dimension of the problem and yield interpretable summary PCA variables. A second-stage logistic regression based on the PCA variables was then applied to obtain the interpretable predictive classification model for MIS-C versus controls. This approach performed well when compared with a supervised ANN-based classification model based on a set of statistically significant variables without principal component adjustment.

## Figures and Tables

**Table 1:** Patient demographics (IQR = interquartile range).

Group	Age in Months Median, [IQR]	Male	Ethnicity (Latino)	White	Black	Total
**COVID-19**	107, [32, 193]	17	9	15	6	34
**MIS-C**	112, [71, 153]	29	19	8	14	48
**KD**	53, [23, 72]	43	25	15	1	62
**Resp-Viral**	34 [15, 91]	16	12	16	4	33
**Controls**	143, [74, 189]	37	17	47	5	69
**Total**	---	142	82	101	30	246

**Table 2:** Eighteen immunological biomarker variables with statistically significant pairwise differences (Tukey method) between MIS-C patients and controls (n = 246). Loadings and percentages of total variation explained shown for first two principal components.

Statistically Significant Biomarkers	MIS-C Versus Controls Adjusted *p*-Value	PCA1 (%Variation = 39.9%)	PCA2 (%Variation = 14.4%)
CCL4	<0.001	0.169	−0.11
CXCL11	<0.001	0.15	−0.322
GMCSF	0.017	0.221	0.315
IL-1β	0.01	0.303	0.304
IL-2	0.006	0.256	0.301
IL-4	<0.001	0.262	−0.226
IL-5	0.02	0.322	0.035
IL-6	<0.001	0.177	−0.336
IL-7	<0.001	0.273	0.262
IL-8	<0.001	0.274	−0.292
IL-10	<0.001	0.147	−0.216
IL-13	<0.001	0.299	−0.124
IL-15	<0.001	0.153	−0.19
IL-18	<0.001	0.143	−0.261
IL-21	<0.001	0.249	0.156
IL-23	0.002	0.299	0.257
IL-33	0.001	0.133	−0.148
TNF-α	<0.001	0.254	−0.067

**Table 3:** Statistically significant biomarkers with high correlations (correlations > 0.5 are in bold).

	GMCSF	IL-1β	IL-2	IL-4	IL-6	IL-7	IL-8	IL-13	IL-21	IL-23	TNF-α
GMCSF	1										
IL-1β	**0.74**	1									
IL2	**0.60**	**0.76**	1								
IL4	0.15	0.36	0.20	1							
IL6	0.01	0.13	0.07	**0.60**	1						
IL7	**0.54**	**0.80**	**0.65**	0.37	0.25	1					
IL8	0.15	0.35	0.18	**0.87**	**0.59**	0.37	1				
IL13	0.29	**0.53**	0.32	**0.91**	**0.52**	0.48	**0.86**	1			
IL21	0.43	**0.56**	**0.69**	0.34	0.17	0.49	0.28	0.38	1		
IL23	**0.56**	**0.79**	**0.67**	0.45	0.45	**0.74**	0.43	**0.62**	**0.59**	1	
TNF-α	0.47	**0.52**	0.41	0.22	0.22	0.43	0.34	0.33	0.35	0.45	1

**Table 4:** Logistic regression classification and diagnostics.

Variables	OR	Standard Error	Test Statistic	*p*-Value	95% Confidence Interval for OR
PCA1	3.45	0.87	4.91	<0.001	[2.11, 5.66]
PCA2	0.33	0.10	−3.64	<0.001	[0.18, 0.60]

Sensitivity = 83%; specificity = 94%; false+ rate = 9%; false– rate = 11%; overall correctly classified = 90%; AUC = 96%.

**Table 5:** Artificial neural network (hidden layers = 18, 36).

	Sensitivity	Specificity	False+	False−	Overall True	AUC
Artificial Neural Network						
**H = 18**	25%	94%	6%	75%	69%	70%
**H = 36**	65%	89%	11%	35%	80%	73%

## Data Availability

Portions of the data presented in this study are currently available in the RADx Data Hub (https://radxdatahub.nih.gov, accessed on 15 December 2024).
